# Single Strain vs Multiple Strain Probiotics: The Clinician's Choice

**DOI:** 10.7759/cureus.86353

**Published:** 2025-06-19

**Authors:** Dhanasekhar Kesavelu, Arunchander Yadav Krishnamurty, Priya A

**Affiliations:** 1 Paediatric Gastroenterology, Apollo Children’s Hospital, Chennai, IND; 2 Clinical Pharmacy, Apollo Hospitals, Chennai, IND

**Keywords:** children, indications, multi-strain probiotic supplement, probiotics, single strain

## Abstract

Background

The intention of optimizing the intestinal microbial milieu has heightened the interest of incorporating probiotics into nutritional supplements for children and adults as a therapeutic or preventive option for a variety of diseases. Prescriptions of probiotics for multiple clinical indications in paediatrics have substantially increased in the past decades and continue to rise due to convincing and growing scientific evidence around them.

Aim

To prospectively analyse the probiotic prescription patterns of pediatricians in a tertiary care hospital.

Method

This was a prospective, observational study conducted at a single center involving children aged 0 to 16 years. The study included 53 pediatricians and analyzed 22,500 prescriptions obtained from electronic medical records (EMR).

Results

A total of 22,500 pediatric prescriptions were analyzed, out of which 17,370 (77.20%) were single-strain probiotic prescriptions. Among single-strain probiotics, *Bacillus clausii* (48.57% [8,437/17,370]) was the most prescribed single-strain probiotic that was chosen by the clinician. On the other hand, the most commonly prescribed multi-strain probiotics prescriptions included a combination of *Streptococcus faecalis, Clostridium butyricum, Bacillus mesentericus,* and *Lactobacillus sporogenes* (37.85% [1,656/4,375]).

Conclusion

The study showed a greater preference pattern for single-strain probiotics, particularly Enterogermina among pediatricians.

## Introduction

The human microbiome has a significant influence on the protection and development of a healthy and balanced immune system, fosters cognitive and brain development, and plays a crucial role in maintaining the physiological processes of the host. The intention of optimizing the intestinal microbial milieu has heightened the interest of incorporating probiotics into nutritional supplements for children and adults as a therapeutic or preventive option for a variety of diseases. The term probiotic was defined in 2001 by an expert panel convened by the Food and Agriculture Organization of the United Nations (FAO) and the World Health Organization (WHO) as “live microorganisms which, when administered in adequate amounts, confer a health benefit on the host” [1].

Probiotics have been promoted for the prevention and treatment of several illnesses, with substantial data supporting their effectiveness in certain clinical situations, including gastrointestinal disorders (such as gastroenteritis, intestinal neoplasia, inflammatory bowel disease, and colitis) and non-gastrointestinal diseases, including the treatment and prevention of atopic eczema [2]. In addition, the utilization of probiotics in children has significantly increased in the past few decades, particularly for conditions such as acute gastroenteritis, antibiotic-associated diarrhoea (AAD), *Helicobacter pylori *eradication, and functional gastrointestinal disorders like infantile colic [3-6]. Multiple organizations, including the World Gastroenterology Organization (WGO) [7], European Society of Pediatric Gastroenterology Hepatology and Nutrition (ESPGHAN) [8], American Gastroenterological Association (AGA) [9], International Scientific Association for Probiotics and Prebiotics (ISAPP) [10], and Indian Society of Pediatric Gastroenterology Hepatology and Nutrition (ISPGHAN) [11], approve their use in pediatric care.

Probiotics are categorized into two types based on their composition: single-strain and multi-strain probiotics. These two are frequently a subject of discourse among physicians over their efficacy and safety for children. Single-strain probiotics, as indicated by their name, consist of a singular form of probiotic, frequently recommended for their simplicity and specific efficacy, while multi-strain probiotics are believed to offer enhanced benefits via synergistic actions. However, concerns about potential strain antagonism in multi-strain formulations complicate clinical decision-making [3, 12], which has been described already as “mutual antagonism” [13].

McFarland et al. demonstrated in their study that the efficacy of probiotics is both strain-specific and disease-specific [14], indicating that single-strain probiotics exhibit greater favorable effects due to their targeted specificity for particular diseases. This hypothesis was further corroborated by a meta-analysis performed by McFarland again in 2020. The meta-analysis included 65 randomized-controlled trials (RCTs) (41 involving single strains, 22 involving multi-strain mixtures, and 2 comparing single strains to mixture arms). The results suggested that single-strain probiotics were equivalent to mixtures, and in the majority of instances, multi-strain probiotics did not demonstrate significantly greater efficacy than single-strain probiotics [13]. Though some of the clinical evidence has shown the effectiveness of a multi-strain prebiotic combination against a variety of diseases, the limited number of research leaves this information ambiguous or unrewarded [15].

These differences in efficacy and reliability emphasize the need for evidence-based prescribing practices to ensure optimal outcomes among children. Hence, the primary objective of our study was to prospectively examine the prescribing patterns of practicing pediatricians about probiotics over 15 months, with a focus on the selection of probiotics and including the preference between single-strain and multi-strain products.

## Materials and methods

Study design

This was a prospective, observational, single-center study conducted to analyse the prescribing patterns of probiotics (prescribed for children aged 0-16 years) among pediatricians. The study was carried out at Apollo Children’s Hospital in Chennai (India) with an annual footfall of over 1 million pediatric patients, offering a large patient cohort to evaluate changes in probiotic prescriptions. The main objective of the study was to analyze how pediatricians prescribe probiotics over a 15-month period, with a particular emphasis on the selection of probiotic formulations and the preference of strains. The study intended to investigate the prescription patterns of probiotics, including trends in the types of probiotics administered (e.g., single-strain vs multi-strain) and to identify the most commonly prescribed probiotic during the study period.

Study population

The study included approximately 53 pediatricians who were actively prescribing probiotics for various clinical indications. Prescriptions for children aged 0-16 years were included in the study, and clearly defined probiotic formulations, such as sachets, syrups, capsules, and oral drops, were used. Prescription records that were incomplete or duplicates were excluded from the study in order to ensure data integrity and accuracy. This yielded a final dataset of 22,500 distinct prescriptions obtained from the hospital's electronic medical records (EMR). These prescriptions were gathered over 15 months. The data was entered by pharmacists using a standardized data entry approach to ensure consistency and reduce the possibility of bias. Notably, no direct patient involvement happened for data collection, reducing the possibility of influencing clinical decision-making.

Ethical approval

This study was conducted in accordance with the ethical standards established by the hospital’s Institutional Ethics Committee. Ethical permission was obtained (application no: AMH-C-S-160/09-24), and consent of the clinicians was taken.

Data collection and analysis

The data was collated from pharmacy prescriptions, which were extracted by pharmacists as explained above. The extracted data was collected in Microsoft Excel (Microsoft Corp., Redmond, WA, USA). After collecting the data, descriptive statistical analysis was carried out using Microsoft Excel.

## Results

A total of 22,500 pediatric prescriptions were examined, out of which 17,370 (77.20%) were single-strain probiotic prescriptions. Data indicated that 15 probiotics were regularly given to children, of which five (N = 5) were multiple-strain probiotics (33.33% [5/15]), while the remaining 66.66% (10/15) were single-strain probiotics. Among single-strain probiotics, Enterogermina® (*Bacillus clausii*) (48.57% [8437/17,370]) was the most prescribed single-strain probiotic that was chosen by the clinician. The prescribed probiotics were observed to be of different formulations, including dry syrup, capsule, sachet, and oral drops.

Prescription patterns and preferences of single-strain probiotics

As discussed above, Enterogermina (*Bacillus clausii*) emerged as the most frequently prescribed single-strain probiotic (48.57%), followed by BioGaia Protectis Baby Drops (*Lactobacillus reuteri DSM 17938*, 5ml) (23.87% [4147/17,370]), and Econorm Sachet (*Saccharomyces boulardii*, 250mg) (18.65% [3240/17,370]). Probiotics like ProGG 1 billion CFU oral drops 4ml (*Lactobacillus rhamnosus*), Vizylac lady cap 15's (Lactic acid bacillus), Protectis BioGaia chewable tablets 30's (*L. Reuteri DSM* 17938), Vizylac GG 1 billion 3.5ml (*Lactobacillus rhamnosus GG*), Activkids Uno Biotics Junior S/R (*L. rhamnosus GG*), Bifilac GG vanilla flavour sachet *(L. rhamnosus GG*), and Superflora GG capsule 0.75g (*L. rhamnosus GG*) show relatively lower prescription rates, displaying a less widespread use (Table 1).

**Table 1:** Frequency of single-strain probiotic prescriptions

S. No.	Product name	Frequency of prescriptions (100% [N=17,370])
	Enterogermina	48.57 (8437)
	BioGaia Protectis Baby Drops	23.87 (4147)
	Econorm Sachet 250mg	18.65 (3240)
	ProGG 1 billion CFU oral drops 4ml	6.17 (1072)
	Vizylac Lady Cap 15's	1.28 (223)
	Protectis BioGaia Chewable Tablets 30’s	0.54 (94)
	Vizylac GG 1 billion CFU Drops 3.5ml	0.44 (76)
	Activkids Uno Biotics Junior S/R	0.29 (50)
	Bifilac GG Vanilla Flavour Sachet	0.16 (28)
	Superflora GG Cap 0.75g	0.02 (3)

Prescription preferences of multi-strain probiotics

On the other hand, the most commonly prescribed multi-strain probiotics prescriptions included Bifilac capsules 10's (containing *Lactobacillus acidophilus, Lactobacillus rhamnosus, Bifidobacterium bifidum, and Streptococcus thermophilus*) at 37.85% (1656), followed by Bifilac GG duo capsules 10's (containing *L. rhamnosus GG, B. bifidum, and S. thermophilus*) at 27.70% (1212). Other multi-strain prescribed probiotics included VSL 3 capsule 10's (consisting of *Lactobacillus acidophilus, Lactobacillus plantarum, Lactobacillus casei, Lactobacillus delbrueckii subspecies bulgaricus, Bifidobacterium breve, Bifidobacterium longum, Bifidobacterium infantis*, and *Streptococcus salivarius subspecies thermophilus*), Bifilac 50ml dry syrup (with *L. acidophilus, L. rhamnosus, B. bifidum, and S. thermophilus*), Providac capsules (containing *L. acidophilus LA-5* and *Bifidobacterium BB-12*) (Table 2).

**Table 2:** Frequency of multi-strain probiotic prescriptions

S. No.	Product name	Frequency of prescriptions (100% [N=4,375])
	Bifilac Cap 10's	37.85 (1656)
	Bifilac GG Duo Cap 10's	27.70 (1212)
	VSL 3 Cap 10's	14.63 (640)
	Bifilac 50ml Dry Syrup	14.29 (625)
	Providac caps	5.53 (242)

**Figure 1:**
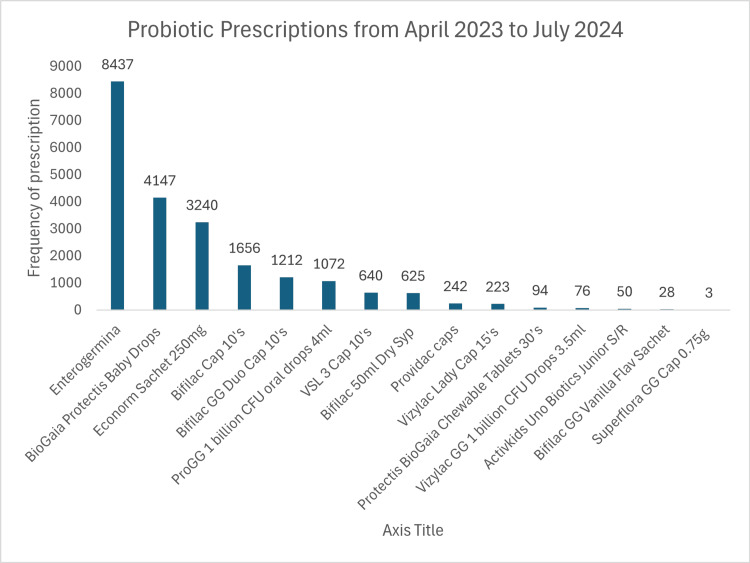
Probiotic Prescriptions from April 2023 to July 2024

Figure 1 demonstrates the complete prescription patterns of single-strain and multi-strain probiotics from April 2023 to July 2024.

## Discussion

There is very limited clinical evidence available to analyse the prescription patterns of probiotics for children. However, the results of our study showed that clinicians preferred single-strain probiotics in children. In addition, *Bacillus clausii* (oxacillin/chloramphenicol resistant [O/C], streptomycin resistant [SIN], tetracycline resistant [T], and neomycin/rifampicin resistant [N/R]) is the largest single-strain probiotic prescribed by practicing pediatricians, indicating its widespread acceptability by clinicians.

Numerous single-strain studies provide evidence in support of recommendations for *B. clausii*. Research indicates that *B. clausii* probiotic formulations are efficient in treating acute diarrhea and are often safe [16, 17]. In addition, *Bacillus* spores remain stable in the extreme gut environment, like acidic pH and the presence of bile salts. Furthermore, the resilience of these spores facilitates their management during storage and medicinal formulations [18, 19].

A study conducted by Kesavelu et al. reported that *B. clausii* was the most effective probiotic strain in reducing diarrhea duration, time to first formed stool, reducing hospital admissions, and earlier recovery compared to other strains such as *Saccharomyces boulardii* and *Lactobacillus rhamnosus GG* [20]. In addition, certain strains of these species are resistant to antibiotics and found to be effective in combined antibiotic-probiotic therapy to maintain a healthy gut environment [21, 22].

Other single-strain probiotics, such as *L. rhamnosus* *GG *and *S. boulardii,* have demonstrated significant efficacy in preventing AAD and reducing the incidence of diarrhea in children [23]. Similarly, *S. boulardii* has demonstrated better efficacy in reducing adverse events related to *H. pylori* eradication therapy [24]. *L. reuteri*, a single-strain probiotic, is also proven effective in children with infantile colic. A clinic-based real-world study conducted by Wadhwa et al. reported that supplementation of *L. reuteri DSM 17938* in infantile colic subjects resulted in a significant reduction of unexplained fussiness and excessive crying [25]. However, in a randomized controlled trial conducted by Canani et al., multi-strain probiotics demonstrated superior efficacy in reducing the duration of diarrhea and hospitalization when compared to single-strain probiotics, including* L. rhamnosus GG*, when combined with oral rehydration solutions [26].

Comprehensive studies of gastrointestinal disorders also indicated that the efficacy of both single- and multi-strain probiotics varies, based on the specific condition and strain used. A study conducted on preterm infants showed that both single and multi-strain probiotics effectively reduced gut dysbiosis, although clinical outcomes, such as time to full feeds, were comparable between groups [27]. According to the most recent ESPGHAN position paper, certain probiotic strains such as *L. rhamnosus GG* and mixed strains of *B. infantis, B. lactis*, and *S. thermophilus* are conditionally advised for preterm infants to help minimize NEC, as long as strict safety and product quality requirements are fulfilled [28]. Interestingly, multi-strain probiotics demonstrated better outcomes in necrotizing enterocolitis (NEC) among preterm infants and gastrointestinal complications [13]. A Bayesian network meta-analysis that evaluated 29 RCTs from low- and middle-income countries (LMICs) reported that a combination of *Bifidobacterium* and *Lactobacillus* strains decreased sepsis, mortality, and NEC. However, these results were limited due to a lesser number of head-to-head strain comparisons [29]. Similarly, single-strain formulations proved to be particularly effective for targeted symptoms such as diarrhea and *H. pylori* treatment [13].

The significant preference towards single-strain probiotics corresponds with growing scientific data supporting their efficacy, safety, and reliability in treating pediatric gastrointestinal disorders, such as acute gastroenteritis and antibiotic-associated diarrhea. This is consistent with global guidelines from organizations such as ESPGHAN, which advocate for the use of single-strain probiotics for specific clinical needs [8]. Due to potential antagonistic interactions across strains [30] that may diminish their efficiency, multi-strain probiotics are recommended at a somewhat lower frequency.

The lack of age-wise stratification and clinical indication data, the use of simple descriptive statistics without comparison analysis, the possibility of selection bias due to the single-center design, and the potential impact of commercial interests on prescription patterns are some of the possible limitations of the study. These variables limit the results to their interpretation and generalizability.

## Conclusions

In conclusion, the findings of this study reveal a clear preference among pediatricians for single-strain probiotic formulations, with *B. clausii* emerging as the most commonly prescribed probiotic strain. This preference underscores a broader trend within pediatric clinical practice favoring single-strain probiotics, particularly in the management of gastrointestinal disorders. Such a pattern likely reflects growing confidence in the clinical efficacy, safety profile, and well-documented strain-specific benefits of these formulations. Importantly, the results also emphasize the critical need for evidence-based prescribing practices in the pediatric population, given the variability in probiotic strains and formulations currently available.
